# Oxidative stress, anti-oxidants and the cross-sectional and longitudinal association with depressive symptoms: results from the CARDIA study

**DOI:** 10.1038/tp.2016.5

**Published:** 2016-02-23

**Authors:** C N Black, B W J H Penninx, M Bot, A O Odegaard, M D Gross, K A Matthews, D R Jacobs

**Affiliations:** 1 Department of Psychiatry, EMGO Institute for Health and Care Research, VU University Medical Center, Amsterdam, The Netherlands; 2 Department of Epidemiology, School of Medicine, University of California, Irvine, Irvine, CA, USA; 3 Department of Laboratory Medicine and Pathology University of Minnesota Medical School, University of Minnesota, Minneapolis, MN, USA; 4 Department of Psychiatry, University of Pittsburgh School of Medicine, Pittsburgh, PA, USA; 5 Division of Epidemiology and Community Health, School of Public Health, University of Minnesota Medical School, University of Minnesota, Minneapolis, MN, USA

## Abstract

Depression may be accompanied by increased oxidative stress and decreased circulating anti-oxidants. This study examines the association between depressive symptoms, F2-isoprostanes and carotenoids in a US community sample. The study includes 3009 participants (mean age 40.3, 54.2% female) from CARDIA (Coronary Artery Risk Development in Young Adults). Cross-sectional analyses were performed on data from the year 15 examination (2000–2001) including subjects whose depressive symptoms were assessed with the Center for Epidemiologic Studies Depression Scale (CES-D) and had measurements of plasma F2-isoprostanes (gas chromatography/mass spectrometry) or serum carotenoids (high-performance liquid chromatography). Carotenoids zeaxanthin/lutein, β-cryptoxanthin, lycopene, α-carotene, β-carotene were standardized and summed. Longitudinal analyses were conducted using the data from other examinations at 5-year intervals. Cross-lagged analyses investigated whether CES-D predicted F2-isoprostanes or carotenoids at the following exam, and vice versa. Regression analyses were controlled for sociodemographics, health and lifestyle factors. F2-isoprostanes were higher in subjects with depressive symptoms (CES-D⩾16) after adjustment for sociodemographics (55.7 vs 52.0 pg ml^−1^; Cohen's *d*=0.14, *P*<0.001). There was no difference in F2-isoprostanes after further adjustment for health and lifestyle factors. Carotenoids were lower in those with CES-D scores ⩾16, even after adjustment for health and lifestyle factors (standardized sum 238.7 vs 244.0, Cohen's *d*=−0.16, *P*<0.001). Longitudinal analyses confirmed that depression predicts subsequent F2-isoprostane and carotenoid levels. Neither F2-isoprostanes nor carotenoids predicted subsequent depression. In conclusion, depressive symptoms were cross-sectionally and longitudinally associated with increased F2-isoprostanes and decreased carotenoids. The association with F2-isoprostanes can largely be explained by lifestyle factors, but lower carotenoids were independently associated with depressive symptoms.

## Introduction

Depression, besides being a leading cause of poor functioning and disability,^1^ is an independent predictor of the onset of somatic disease.^2^ Relative to individuals without, individuals with depression have a higher risk of developing cardiovascular disease,^3^ obesity,^4^ diabetes,^5^ cancer^6^ and cognitive impairment^7^ and have increased mortality rates.^8^ These associations have been found not only in individuals with major depressive disorder, but also in those with depressive symptoms.^2^ Increased oxidative stress and decreased antioxidant levels may be key mechanisms in this association. Oxidative stress is a complex and dynamic biological process that refers to the damaging effects of reactive oxygen species (ROS). ROS are normal products of aerobic metabolism and the body has a range of antioxidant defenses to protect against their potentially harmful effects. However, when there is either an increase in exposure to (or production of) ROS, or a decrease in anti-oxidants levels, damage may occur to lipids, proteins and DNA resulting in cellular dysfunction and disease, or ultimately cell death.^9^

A recent meta-analysis^10^ demonstrated that oxidative stress levels measured by F2-isoprostanes (reflecting oxidative lipid damage) are increased in persons with major depression and/or depressive symptoms, but the number of studies and their sample sizes were limited and many studies did not account for important potential confounding factors. Oxidative stress is associated with a range of sociodemographic, health and lifestyle factors, for example, socioeconomic status and smoking,^9, 11, 12^ that are also known to be associated with depression.^4, 13, 14, 15^ If taken into account, these factors could considerably influence the estimate of the association.

There is also evidence suggesting that anti-oxidants are decreased in depression, illustrated by lower antioxidant levels,^16^ including carotenoids,^17, 18^ and antioxidant enzymes in persons with depressive symptomatology.^19^ Measuring oxidative stress *in vivo* is challenging; levels of ROS are not easily determined owing to their short half-life and highly reactive nature. Alternative approaches include measurements of oxidative damage to protein, lipids or DNA or of levels of antioxidants.

F2-isoprostanes are currently considered to be the marker of choice for oxidative lipid damage.^20, 21, 22, 23^ F2-isoprostanes are formed solely through oxidative processes; this quality and the chemical stability of these compounds make them more reliable markers than other widely used measures of oxidative lipid damage, such as malondialdehyde or thiobarbituric reactive substances, that have limited validity due to the likelihood of artefactual formation.

Carotenoids have antioxidant capacity. This property may contribute to observational studies finding higher carotenoid levels to be associated with reduced risks of metabolic syndrome,^24, 25^ diabetes mellitus,^26, 27, 28^ cardiovascular disease^29, 30^ and cancer.^31^ In humans, the most important carotenoids include β-carotene, lycopene, zeaxanthin/lutein and β-cryptoxanthin. They owe their potent antioxidant action to their ability to quench ROS.^32^ As highly lipophilic molecules, they are located in the lipid bilayer of the cell membrane where they protect against lipid peroxidation.^33^ Studying carotenoids in unison with the F2-isoprostanes provides insight into two important and interdependent aspects of redox homeostasis.

This study describes the cross-sectional associations of depressive symptoms with F2-isosprostanes and carotenoids in large community samples, taking into account a wide range of important sociodemographic, health and lifestyle factors. In particular, this study includes data on dietary patterns, which is not available for the majority of previous research. Dietary patterns may be an important potential confounding factor in the association with depression; dietary intake is the sole source of carotenoids in humans and F2-isoprostanes have previously been demonstrated to be associated with dietary pattern independently of other health and lifestyle factors.^34^ In addition, this study examines the relationships between F2-isosprostanes, carotenoids and depressive symptoms over multiple time points to gain insight into the temporal directionality of the association as it is unclear whether oxidative stress leads to depressive symptoms or vice versa. It is possible that depressive symptoms lead to unhealthy behaviors that in turn increase exposure to oxidative stress. Alternatively, increased oxidative stress, to which the brain is particularly vulnerable, may cause oxidative damage, making an individual susceptible to developing depressive symptoms.^35^

This report comprises, to our knowledge, the largest sample in which the association of depressive symptoms, F2-isosprostanes and carotenoids have been examined together.

## Materials and methods

### Study sample

Data are from the CARDIA (Coronary Artery Risk Development in Young Adults) study and the ancillary YALTA (Young Adult Longitudinal Trends in Antioxidants) study. CARDIA is a longitudinal, multicenter epidemiological study on the development of risk factors for cardiovascular disease. The study design and recruitment of participants have been described elsewhere.^36^ In brief, from 1985 to 1986, subjects aged 18–30 years were recruited from four sites (Birmingham, AL; Chicago, IL; Minneapolis, MN; and Oakland, CA) in the United States. The sampling strategy resulted in a cohort balanced by race (52% black), sex (55% female) and education (40% with <12 years of education) comprising 5115 individuals at baseline. Follow-up assessments were conducted after 2, 5, 7, 10, 15, 20 years from the baseline assessment with retentions rates of 91%, 86%, 81%, 79%, 74%, 72% of the surviving cohort, respectively. YALTA measured serum carotenoids at years 0, 7 and 15 and plasma F2-isoprostanes at years 15 and 20. Institutional review committee approval was obtained from each site and written informed consent was obtained from the participants for all assessments.

**Figure 1:**
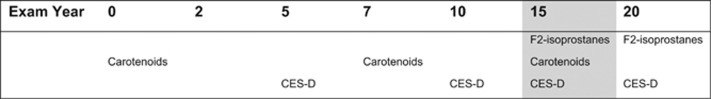
Available data on F2-isoprostanes, carotenoids and CES-D in CARDIA exam years 0–20. CARDIA, Coronary Artery Risk Development in Young Adults; CES-D, Center for Epidemiologic Studies Depression Scale.

The cross-sectional analyses in this study were conducted with data from the year 15 CARDIA assessment, as this is the only time point that allowed analysis of both F2-isoprostanes and carotenoids (see Figure 1) with depressive symptoms. The sample comprised 3009 participants for whom data were available on depressive symptoms measured by the Center for Epidemiologic Studies Depression Scale (CES-D) and data on either F2-isoprostanes (*n*=2974) or carotenoids (*n*=2889). For the longitudinal analyses, data were used on F2-isoprostanes from years 15 and 20, carotenoids from baseline and years 7 and 15, and CES-D from years 5, 10, 15 and 20.

### Depressive symptoms

Depressive symptoms were assessed using the 20-item CES-D.^37^ Subjects indicate on a four-point scale how often they experienced a symptom in the past week. Four items relating to positive affect are reverse scored and the overall score is a sum of the responses across the 20 items (possible range 0–60). A cutoff score of ⩾16 indicates a clinically significant depressed mood. The CES-D has been found to have good internal consistency and adequate test–retest reliability. Construct validity of the scale is supported by correlations with other self-report measures, clinical ratings of depression, and clinical interviews.^38^

CES-D scores were analyzed in four different ways. First, total CES-D scores were analyzed as a continuous measure. Second, the CES-D was used as a categorical measure using the cutoff score ⩾16. Third, a categorical measure based on CES-D scores and antidepressant use comparing subjects with CES-D⩾16 and/or current antidepressant to subjects with CES-D<16 and no antidepressant use was created. (Data on antidepressant use was obtained through a structured interview assessed at the same examination point.) Finally, to study the impact of exposure to chronic depressive symptoms, a count variable was created representing the number of times the CES-D was ⩾16 based on the assessments at years 5, 10 and 15. Subjects were compared with those who never had a CES-D score ⩾16.

### Oxidative stress and anti-oxidants

All the participants were instructed to adhere to an overnight fast and were asked to avoid smoking and heavy physical activity for at least 2 h before blood collection at each examination. After serum and plasma separation from whole blood, aliquots were stored at −70 °C until they were shipped on dry ice to a central laboratory.

#### Plasma F2-isoprostanes

YALTA used plasma obtained at CARDIA years 15 and 20 to measure F2-isoprostanes with a gas chromatography–mass spectrometry-based method^39^ by the Molecular Epidemiology and Biomarker Research Laboratory at the University of Minnesota (Minneapolis, MN, USA), as previously described.^40^ All the samples were analyzed within 1 year of collection. Substudies demonstrated stability of F2-isoprostanes (no *ex vivo* loss or formation) during blood collection and processing procedures. Analytical variation of the method was 10% for each of three control pools assembled in 2000 and assayed repeatedly between October 2000 and August 2007; the values of these control pools were stable over time, implying that both assay and stored samples were stable.^34^ Thus, year 15 and 20 plasma F2-isoprostane concentrations are directly comparable.

#### Serum carotenoids

YALTA used sera obtained at CARDIA years 0, 7 and 15 to assay the carotenoids, α- and β-carotene, lycopene, zeaxanthin/lutein and β-cryptoxanthin (Molecular Epidemiology and Biomarker Research Laboratory, University of Minnesota), with an HPLC-based assay modified from the method of Bieri *et al.*^41^ to optimize detection of carotenoids with calibration as described by Craft *et al.*^42^ and sample handling as described by Gross *et al.*^43^ Calibration was performed with pure compounds (Hoffmann-La Roche, Basel, Switserland; Sigma Chemical (now Sigma-Aldrich, St Louis, MO, USA)). Quality-control procedures included routine analysis of plasma and serum control pools containing high and low concentrations of each analyte. In addition, the laboratory routinely analyzed NIST reference sera and was a participant in the NIST Fat-Soluble Vitamin Quality Assurance Group. The coefficients of variance were <10% for all analytes and control pools. The intra-class correlation coefficients (between-person variance/between-person plus within-person variance) were 0.93 for α-carotene, 0.98 for β-carotene, 0.73 for zeaxanthin/lutein, 0.97 for β-cryptoxanthin and 0.73 for lycopene.^44^

From a dietary and physiological perspective, the five carotenoids (zeaxanthin/lutein, β-cryptoxanthin, lycopene, α- and β-carotene) are closely interconnected and were therefore analyzed as total carotenoids (sum of five carotenoids) and as well as individually, similar to previous reports on carotenoids in this sample^45^ and previous studies on carotenoids and depressive symptoms.^46^

### Covariates

#### Demographic variables

Participants' age, sex and race were assessed by self-report. Education was assessed by asking participants to report their highest level of education.

#### Number of somatic diseases

Participants reported whether they had ever been diagnosed with any of twenty-seven major or chronic health conditions, and whether they had had these conditions in the past year (see footnote 'd' in Table 1). The total number of self-reported diagnoses of diseases served as the covariate.

#### Supplement use

Participants were asked whether they had used any vitamin or mineral supplements in the past year as part of the CARDIA Diet Practices, Behaviors and Attitudes Questionnaire. Those using a multivitamin, vitamin A, C, E, beta-carotene or an antioxidant combination were defined as supplement users.

#### Diet quality

Diet was assessed at years 0, 7 and 20 through an interviewer-administered validated diet history in which participants were asked open-ended questions about their dietary pattern in the last month. Food groups were classified as beneficial (*n*=20), adverse (*n*=13) or neutral (*n*=13) in terms of hypothesized health effects. This score (*A Priori* Diet Quality Score) has been previously described in more detail.^34^ The theoretical range of score is 0–132 with higher scores indicating higher hypothesized diet quality. This diet quality score is characterized by long-term stability. Correlation coefficients for dietary scores at years 0 and 7, and years 7 and 20 were 0.65 and 0.64, respectively. As dietary data are not available for the year 15 assessment, the mean of the year 0, 7 and 20 diet scores was calculated based on as many of the three data points as were available.

#### Smoking status

Self-reported smoking was classified as nonsmoker (never and former) or current smoker.

#### Alcohol consumption

Participants were categorized as non-drinker, moderate drinker (⩽14 units per week for males and ⩽7 units per week for females) or heavy drinker (>14 units per week for males and >7 units per week for females).

#### Physical activity

A validated interview-administered questionnaire (CARDIA Physical Activity History, a simplified version of the Minnesota Leisure Time Physical Activity Questionnaire^47^) was used to assess physical activity in the past year, from which exercise units were calculated.

#### Body mass index

Body mass index (BMI) was calculated as weight (kg) divided by height squared (m^2^). Height and weight were recorded to the nearest 0.5 cm and 0.2 kg.

All scales used to assess health behaviors are accessible on http://www.cardia.dopm.uab.edu/.

### Statistical analyses

Statistical analyses were performed using SPSS version 20.0 (IBM, Armonk, NY, USA). Independent *t*-tests were used to compare means of continuous variables and chi-square tests to compare categorical variables between persons with and without depressive symptoms. To analyze total carotenoids, a sum score of the standardized values (*t*-scores) of the five carotenoids was created. F2-isoprostanes and the sum of five carotenoids were log-transformed for the analysis.

Linear regression examining the cross-sectional association between F2-isoprostanes or carotenoids (dependent variables) and CES-D scores (main predictor) were conducted with three models. Model 1 was adjusted for sociodemographics (age, sex, race and education); model 2 was additionally adjusted for supplement use and the number of somatic diseases; model 3 was additionally adjusted for health and lifestyle factors, including diet, BMI, smoking, alcohol consumption and physical activity. All the models were adjusted for research center (Birmingham, AL; Chicago, IL; Minneapolis, MN or Oakland, CA). Analysis of covariance was used to calculate the mean levels of F2-isoprostanes and carotenoids in those with and without depressive symptoms (CES-D cutoff ⩾16). Reported levels were back-transformed to geometric means. Effect's sizes Cohen's *d*^48^ were calculated on the basis of the means, standard deviations and number of subjects.

To demonstrate which health and lifestyle factors had the greatest effects on the association between F2-isoprostanes/carotenoids and CES-D scores, change of estimate analyses were conducted. Models were created for each health and lifestyle factor by adding them to the model already adjusted for sociodemographics (model 1). The percentage of change in standardized regression coefficient of the CES-D score after the addition of a health and lifestyle factor was calculated for each covariate.

To determine whether depressive symptoms predict oxidative stress levels over time (or vice versa), cross-lagged linear regression analyses were conducted. F2-isoprostane levels at year 20 (dependent variable) and CES-D at year 15 (main predictor) were adjusted for covariates at year 15 and F2-isoprostanes at year 15. Similarly, analyses were conducted with CES-D at year 20 (dependent variable) and F2-isoprostanes at year 15 (main predictor), adjusted for covariates at year 15 and CES-D at year 15. For the sum of the five carotenoids at year 15, cross-lagged analyses were conducted with CES-D at year 10 as the main predictor, with covariates at year 10, additionally adjusted for carotenoids at year 7 (no data were available on carotenoids at year 10). Finally, analyses were conducted with CES-D at year 20 (dependent variable) and the carotenoids at year 15 (main predictor), adjusted for covariates at year 15 and CES-D at year 15.

## Results

### Sample characteristics

The sample was 54% female, 55% white and the mean age was 40.3 years (s.d. 3.6). The mean CES-D score was 8.9 (s.d. 7.7) with 15.7% scoring ⩾16 and 20.0% scoring ⩾16 and/or currently using antidepressants. Those with a CES-D score ⩾16 were more likely to be female, black and have less than high school education. They differed significantly from those with CES-D score lower than 16 on all health and lifestyle factors, except supplement use. Those with depressive symptoms were more likely to be smokers, heavy drinkers, have a higher BMI, have somatic diseases, have a lower diet quality score and to be less physically active (Table 1). F2-isoprostanes and the summed carotenoids were significantly inversely correlated (Pearson's rho: −0.27, *P*<0.001). Table 2 shows the associations of these markers with the sociodemographic, health and lifestyle covariates. (See Supplementary Table 1 for the results of the individual carotenoids.)

### F2-isoprostanes

#### Cross-sectional analyses

**Figure 2:**
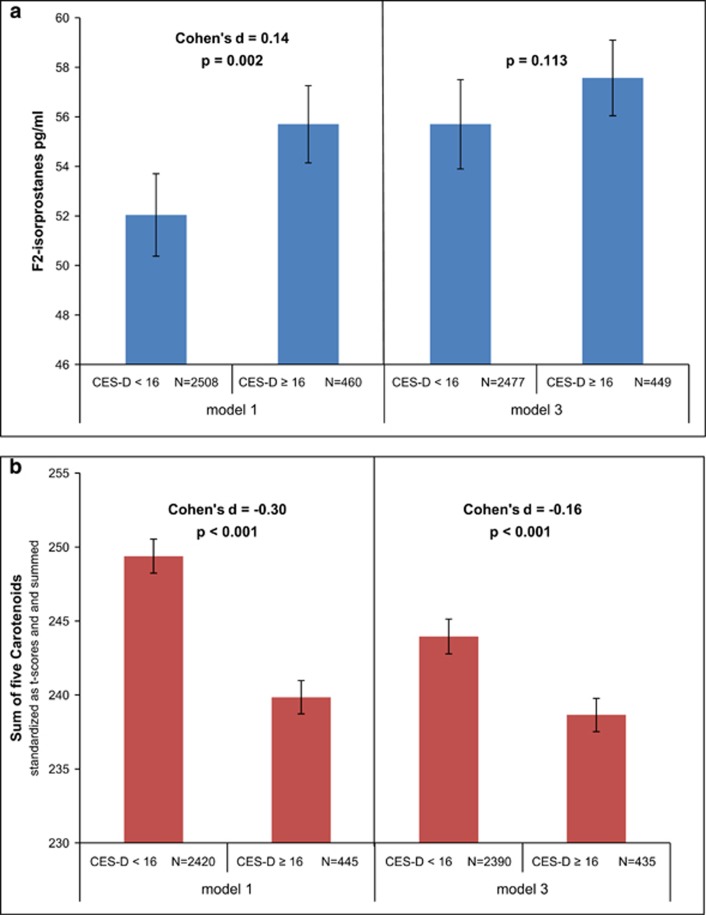
Mean levels of F2-isoprostanes (**a**) and carotenoids (**b**) in non-depressed (CES-D<16) and in depressed subjects (CES-D⩾16) adjusted for sociodemographics only (model 1) and adjusted for both sociodemographics and health and lifestyle factors (model 3), CARDIA exam year 15. In **a**, mean levels of F2-isoprostanes in pg ml^−1^. In **b**, mean levels of the sum of five carotenoids, standardized as *t*-scores and summed. Model 1: center, age, sex, race, education. Model 3: center, age, sex, race, education, supplement use, somatic disease, diet, alcohol consumption, smoking, BMI, physical activity. F2-isoprostanes and carotenoids were log-transformed for analysis. Reported levels are back-transformed values. CES-D cutoff score ⩾16, increased depressive symptoms. BMI, body mass index; CARDIA, Coronary Artery Risk Development in Young Adults; CES-D, Center for Epidemiologic Studies Depression Scale.

CES-D (continuous score) was positively associated with F2-isoprostanes after adjustment for sociodemographics (model 1: *β*=0.05, *P*=0.009) and supplement use and somatic disease (model 2: *β*=0.05, *P*=0.010; Table 3). After additional adjustment for lifestyle, there was no association between CES-D and F2-isoprostanes (model 3: *β*=0.01, *P*=0.731). In depressed subjects (CES-D⩾16), the mean F2-isoprostane level was 55.7 pg ml^−1^ (95% CI=53.6–58.0) vs 52.0 pg ml^−1^ (95% CI=51.0–53.1) in the non-depressed after adjustment for sociodemographics (model 1: Cohen's *d*= 0.14, *P*=0.001; Figure 2). There was no significant difference between the groups after adjustment for health and lifestyle factors: mean F2-isoprostane level was 57.5 pg ml^−1^ (95% CI=55.4–59.9) in depressed versus 55.7 pg ml^−1^ in non-depressed subjects (95% CI=54.4–57.0; *P*=0.113; Figure 2a). Change in estimate analyses revealed BMI, smoking and diet had the greatest effects on the association (See Supplementary Table 3).

Depression defined as CES-D⩾16 and/or current antidepressant use was positively associated with F2-isoprostanes after adjustment for sociodemographics (model 1: *β*=0.06, *P*<0.001) and after health and lifestyle (model 3: *β*=0.03, *P*=0.047). The association of F2-isoprostanes and the number of times that CES-D scores were ⩾16 over the years 5, 10 and 15 was significant for those with increased scores two or three times (after adjustment for sociodemographics), illustrating the association was stronger when depressive symptoms had a more chronic course (model 1: CES-D⩾16 two times, *β*=0.05, *P*=0.009; CES-D⩾16 three times, *β*=0.04, *P*=0.033). The association was no longer significant after additional adjustment for health and lifestyle (model 3: CES-D⩾16 two times, *β*=0.03, *P*=0.111; CES-D⩾16 three times, *β*=0.01, *P*=0.603).

#### Cross-lagged longitudinal analyses

Cross-lagged analyses of CES-D and F2-isoprostanes at years 15 and 20 revealed CES-D⩾16 (as well as CES-D⩾16 and/or current antidepressant) at year 15 were associated with higher F2-isoprostanes at year 20 (model 1: *β*=0.05, *P*=0.003; Table 4). These findings remained significant after adjustment for health and lifestyle factors (model 3: *β*=0.03, *P*=0.048). F2-isoprostanes at year 15 were associated with CES-D scores at year 20 after adjustment for sociodemographics (model 1: *β*=0.04, *P*=0.028), but not after further adjustment for health and lifestyle (model 3: *β*=0.03, *P*=0.095).

### Carotenoids

#### Cross-sectional analyses

CES-D (continuous score) was negatively associated with the sum of five carotenoids after adjustment for sociodemographics (model 1: *β*=−0.13, *P*<0.001), and after additional adjustment for supplement use and somatic disease (model 2: β=−0.13, *P*<0.001), and also after adding lifestyle factors (model 3: *β*=−0.07, *P*<0.001; Table 3). In those with depressive symptoms, the mean level of the sum of the five carotenoids was 239.8 (95% CI=237.2–242.7) vs 249.4 (95% CI=248.1–250.9; standardized sum) in those without depressive symptoms after adjustment for sociodemographics (model 1: Cohen's *d*=−0.30, *P*<0.001; Figure 2b) and 238.7 (95% CI=236.0–241.3) vs 244.0 (95% CI=242.5–245.7) after additional adjustment for health and lifestyle factors (model 3: Cohen's *d*=−0.16, *P*<0.001; Figure 2b). Change in estimate analyses revealed that smoking, diet and to a lesser extent BMI had the greatest effects on the association (See Supplementary Table 3). The number of previously measured CES-D⩾16 tended to be related only for those who scored ⩾16 at all three measurements (model 3: *β*=−0.03, *P*=0.062). The pattern of results was similar across the individual carotenoids (see Supplementary Table 2).

#### Cross-lagged longitudinal analyses

Cross-lagged analyses revealed all CES-D variables at year 10 predicted carotenoids at year 15 after adjustment for sociodemographics (CES-D⩾16 model 1: *β*=−0.07, *P*<0.001) as well as after adjustment for health and lifestyle factors (CES-D⩾16 model 3: *β*=−0.05, *P*=0.002). Carotenoids at year 15 were not associated with CES-D at year 20 (model 3: *β*=−0.01, *P*=0.560; Table 4).

## Discussion

Higher F2-isoprostanes and lower carotenoid levels were cross-sectionally associated with increased depressive symptoms. For the F2-isoprostanes this association was largely explained by the lifestyle factors smoking, diet and BMI. For the carotenoids, this association is partially attenuated by these factors but remains present even after controlling for them. Longitudinal analyses show that depressive symptoms predict both the F2-isoprostane and carotenoids levels 5 years later; however, neither marker predicts future depressive symptoms. Overall, the effect sizes found in this study were small in size.^48^

A recent meta-analysis^10^ found increased F2-isoprostanes in persons with major depressive disorder and depressive symptoms. This is in contrast with the results of the present study and may be explained by the fact that several of the studies in this meta-analysis took into account some, but not all of the health and lifestyle factors included in these analyses. Previous large scale studies also reported decreased carotenoid levels in depressive symptoms.^17, 18^ A novel finding in this study is that the association remains present even after controlling for diet quality, which should be considered an important confounder as dietary intake is the sole source of carotenoids. The longitudinal associations, in which depressive symptoms predict future oxidative stress/antioxidant levels, are small in size but significant, despite the conservative method of estimation (correcting for previous levels of F2-isoprostanes or carotenoids).

Our results suggest that health and lifestyle factors are important mechanisms in the association between depressive symptoms and oxidative stress, especially for F2-isoprostanes. The lower carotenoid levels found in depressive symptoms may be a consequence of relatively low intake of foods rich in carotenoids, such as fruits and vegetables; a dietary pattern that has previously been associated with depression.^49, 50^ In this study, however, the association between depressive symptoms and carotenoids was also present after adjusting analyses for a measure of diet quality. Other unhealthy lifestyle behaviors associated with depressive symptoms, such as smoking and alcohol consumption, increase the exposure to oxidative damage, as reflected by increased F2-isoprostane levels.^51^ One of the mechanisms by which smoking may decrease carotenoids levels is through causing an increase in metabolic rate, which in turn increases oxidative stress exposure, leading to a higher expenditure of antioxidant micronutrients such as the carotenoids.^52^ Exposure of human plasma to cigarette smoke has been shown to reduce carotenoids and other antioxidant levels.^53^

There is also evidence suggesting that common genetic factors underlie both low carotenoid levels and depressive symptoms: a recent study demonstrated a single-nucleotide polymorphism associated with low levels of β-cryptoxanthin was also associated with depressive symptoms.^54^

Besides increased exposure to ROS from exogenous sources owing to poor health and lifestyle behaviors, there is evidence to suggest that depression is also accompanied by increased endogenous production of ROS, possibly through mitochondrial dysfunction.^55^ The brain is particularly vulnerable to oxidative damage owing to its large oxygen consumption and relatively weak antioxidant defenses. Sustained oxidative brain damage during a depressive episode may make a sufferer prone to developing another depressive episode. Therefore, it has been hypothesized that exposure to oxidative stress could be an explanatory mechanism in the remitting and chronic course of depressive disorders.^35^

There is some evidence to suggest that antidepressants have antioxidant properties and may act through reducing pro-inflammatory cytokines and ROS production and improving levels of antioxidants such as superoxide dismutase.^56^ A number of studies have demonstrated that successful treatment with antidepressants decreases markers of oxidative stress and/or increases markers of antioxidant activity.^57, 58, 59, 60, 61^ However, these findings are limited and conflicting.^19, 62, 63, 64, 65, 66, 67^ To illustrate, Chung *et al.*^64^ found that serotonin re-uptake inhibitor treatment actually increased F2-isoprostane levels, despite reducing depressive symptoms.

Further understanding of the complex biological processes involved in oxidative stress will be necessary to successfully exploit (anti-)oxidative processes as an avenue for treatment. Even in diseases in which the role of oxidative stress is considered well established (such as cancer), the results of preventive treatment with antioxidants have been disappointing, or even harmful.^68, 69, 70^ The paradigm within which oxidative stress is perceived as damaging and antioxidants as protective is likely not an adequate model to this end. Although previously ROS were thought of purely as damaging to cells, the vital role they play in defense against pathogens and intracellular signaling is now recognized. Similarly antioxidants should not be thought of purely as benevolent factors. The finding that β-carotene supplementation in smokers lead to a significant increase in lung cancer incidence,^71, 72^ is a clinical example of this. Omega-3 fatty acids are among the most widely studied supplements for the treatment of depression and may reduce oxidative stress through their anti-inflammatory properties. A recent meta-analysis of randomized trials found that they indeed reduce depressive symptoms in patients with a diagnosis of major depressive disorder or increased depressive symptoms.^73^ The effect of carotenoid supplements on depression has (to our knowledge) not yet been investigated, but observational data suggest that dietary patterns are associated with depressive symptoms.^74^ The same can be said of smoking, alcohol use, obesity and physical inactivity, all of which occur more frequently in those with depressive symptoms,^4, 13, 14, 15^ and all of which are associated with oxidative stress.^9, 11^ This suggests that addressing these behaviors may be the most effective course of action in depression.

This study has some important limitations. Although the CES-D is a well-established and sensitive tool for assessing depressive symptoms, like most self-report questionnaires for depression it lacks the specificity of diagnosis by a clinician or through a (semi-)structured interview. This may have led to misclassification of some subjects in this study as being depressed. In addition, due to the observational design and the varying availability of oxidative stress, antioxidant and depression data at different time points, the possibilities for examination of the longitudinal associations were limited. The effect sizes are statistically significant but small; this should be considered when interpreting the potential clinical impact of the association. Individual markers of oxidative stress and/or antioxidants cannot and do not fully reflect the ongoing and complex biological process of redox homeostasis. This limitation applies to all human studies in the field of oxidative stress and should be considered in the interpretation of all study results. F2-isoprostanes reflect oxidative lipid damage, but are only one product of lipid peroxidation. Furthermore oxidative damage also affects DNA and proteins, but markers of these processes such as 8-OHdG and protein carbonyls, are not available in this study. Although carotenoids are important potent antioxidants, their levels do not reflect all aspects of the antioxidant defense system, that includes many more enzymatic (for example, superoxide dismutase, catalase) and non-enzymatic antioxidants (ascorbic acid). The main strengths of this study are the large sample size, the measurements of circulating F2-isoprostanes and carotenoids with gold-standard techniques, the ability to control for a wide range of important health and lifestyle confounders, and determination of the temporal associations between depression and F2-isoprostanes and carotenoids.

In conclusion, this study demonstrates that F2-isoprostanes and carotenoids are associated with depressive symptoms, both cross-sectionally and longitudinally, and that health and lifestyle factors are important mechanisms in this association, especially in F2-isoprostanes. Further large scale research on oxidative stress and antioxidants should investigate these associations in individuals meeting the diagnostic criteria for a mood disorder.

## Figures and Tables

**Table 1:** Sample characteristics by depressive symptoms (CES-D< or ⩾16)

*CARDIA exam year 15*		*CES-D<16*	*CES-D⩾16*	P	*Total*
		*(*N=*2538)*	*(*N=*471)*		*(*N=*3009)*^a^
*Oxidative stress*					
F2-isoprostanes pg ml^−1^	Median (IQR)	58.2 (38.2–68.3)	55.4 (41.9–75.0)	<0.001	50.9 (38.5–69.4)
					
*Carotenoids*						
Sum of five carotenoids (*t*-scores)^b^	Median (IQR)	252.0 (228.6–268.5)	234.4 (221.3–252.4)	<0.001	243.4 (227.2–266.50)	
Zeaxanthin/lutein μg dl^−1^	Median (IQR)	25.9 (17.5–31.3)	20.2 (15.6–27.6)	<0.001	23.1 (17.1–30.7)	
β-cryptoxanthin μg dl^−1^	Median (IQR)	12.1 (6.5–15.2)	8.4 (5.8–12.9)	<0.001	9.6 (6.4–14.7)	
Lycopene μg dl^−1^	Median (IQR)	40.2 (27.3–50.4)	35.0 (24.4–46.0)	<0.001	37.7 (26.9–49.7)	
α-carotene μg dl^−1^	Median (IQR)	5.9 (2.0–7.3)	2.6 (1.3.1–4.9)	<0.001	3.7 (1.9–6.9)	
β-carotene μg dl^−1^	Median (IQR)	22.5 (9.5–16.4)	12.1 (7.1–20.1)	<0.001	15.6 (9.1–26.4)	
					
*Depressive symptoms*						
CES-D	Mean (s.d.)	6.3 (4.2)	22.8 (7.0)		8.9 (7.7)	
CES-D⩾16		—	—		15.7%	
CES-D⩾16 and/or antidepressant use		—	—		20.0%	
Antidepressant users		5.1%	19.1%	<0.001	7.3%	
*N* of times CES-D⩾16	0	75.4%	—	<0.001	63.9%	
over years 0, 10 and 15^c^	1	19.3%	29.0%		20.8%	
	2	5.3%	35.0%		9.8%	
	3	—	36.0%		5.5%	
					
*Sociodemographics*						
Age	Mean (s.d.)	40.3 (3.6)	40.1 (3.7)	0.15	40.3 (3.6)	
Female		52.4%	63.9%	<0.001	54.2%	
White		58.3%	37.2%	<0.001	55.0%	
Education				<0.001		
	⩽High school	44.4%	55.9%		46.2%	
	(some) College	43.6%	33.3%		42.0%	
	⩾Master's degree	11.9%	10.9%		11.8%	
					
*Health and lifestyle*						
*N* somatic diseases^d^	Mean (s.d.)	0.9 (1.1)	1.4 (1.5)	<0.001	1.0 (1.2)	
Supplement users^e^		61.4%	62.2%	0.74	61.5%	
Diet quality score^f^	Mean (s.d.)	67 (11)	63 (11)	<0.001	67 (11)	
Smoking	Non (never and former)	81.8%	65.7%	<0.001	79.3%	
	Current	18.2%	34.3%		20.7%	
Alcohol				<0.001		
	♂/♀ 0 units per week	46.5%	50.1%		47.1%	
	♂⩽14/♀⩽7 units per week	42.0%	33.3%		40.7%	
	♂>14/♀>7 units per week	11.5%	16.6%		12.3%	
BMI kg/m^2^	Mean (s.d.)	28.3 (6.0)	29.7 (7.3)	<0.001	28.5 (6.2)	
Physical activity (exercise units)	Mean (s.d.)	367 (290)	281 (252)	<0.001	353 (286)	

Abbreviations: BMI, body mass index; CARDIA, Coronary Artery Risk Development in Young Adults; CES-D, Center for Epidemiologic Studies Depression Scale; IQR, interquartile range; *N*, number.

a Year 15 *N*=3009 with valid CES-D score and valid F2-isoprostane (*N*=2974), at least one carotenoid value (*N*=2889), all five carotenoid values (*N*=2865).

b Sum of standardized values (*t*-scores) of zeaxanthin/lutein, β-cryptoxanthin, lycopene, α-carotene, β-carotene.

c Number of times CES-D⩾16 over assessments at CARDIA exam years 5, 10, 15.

d Number or somatic diseases included in count: high blood pressure, high cholesterol, heart problem, diabetes, hepatitis in the past year, kidney failure/dialysis/transplant in the past year, nephritis in the past year, other kidney disease in the past year, liver cirrhosis, other liver disease in the past year, gallstones in the past year, migraine in the past year, peripheral vascular disease, cancer (ever), thyroid disease (ever), ulcer in the past year, other digestive disease in the past year, gout in the past year, asthma in the past year, epilepsy with seizures in the past year, tuberculosis in the past year, emphysema in the past year, multiple sclerosis in the past year, stroke in the past year, chronic bronchitis in the past year, HIV (ever), blood clot (past year), other major disease, polycystic ovarian syndrome.

e Use of a multivitamin, vitamin A, C, E, beta-carotene or an antioxidant combination.

f Average score over CARDIA exam years 0, 7 and 20.

**Table 2:** Cross-sectional univariate associations of F2-isoprostanes and carotenoids with covariates^a^

*CARDIA exam year 15*		*F2-isoprostanes*		*Sum of five carotenoids*^b^	
		β	P	β	P
*Depressive symptoms*					
CES-D		0.07	<0.001	−0.16	<0.001
CES-D⩾16		0.08	<0.001	−0.14	<0.001
CES-D⩾16 and/or antidepressant use		0.09	<0.001	−0.13	<0.001
Antidepressant users		0.07	<0.001	−0.06	<0.001
*N* of times CES-D⩾16	0	Ref.		Ref.	
over years 0, 10 and 15^c^	1	0.06	0.006	−0.05	0.007
	2	0.07	<0.001	−0.09	<0.001
	3	0.06	0.003	−0.09	<0.001
					
*Sociodemographics*						
Age		−0.02	0.180	0.04	0.023	
Sex	(male reference)	0.27	<0.001	0.00	0.984	
Race	(white reference)	0.00	0.820	−0.13	<0.001	
Education	⩽High school	Ref.		Ref.		
	(some) College	−0.09	<0.001	0.23	<0.001	
	⩾Master's degree	−0.03	0.137	0.08	<0.001	
					
*Health and lifestyle*						
*N* somatic diseases^d^		0.07	<0.001	−0.07	<0.001	
Supplement users^e^		−0.13	<0.001	0.12	<0.001	
Diet quality score^f^		−0.11	<0.001	0.33	<0.001	
Smoker	Non (never and former)	Ref.		Ref.		
	Current	0.08	<0.001	−0.23	<0.001	
Alcohol	♂/♀ 0 Units per week	Ref.		Ref.		
	♂⩽14 /♀⩽7 Units per week	−0.10	<0.001	0.10	<0.001	
	♂>14/♀>7 Units per week	0.07	<0.001	−0.03	0.078	
BMI kg/m^2^		0.34	<0.001	−0.28	<0.001	
Physical activity	Exercise units (EU)	−0.16	<0.001	0.13	<0.001	

Abbreviations: BMI, body mass index; CARDIA, Coronary Artery Risk Development in Young Adults; CES-D, Center for Epidemiologic Studies Depression Scale; *N*, number; Ref., reference.

a F2-isoprostanes and carotenoids log-transformed for linear regression analysis. Results are reported as standardized regression coefficients. All the results are adjusted for CENTER at baseline.

b Sum of standardized values (*t*-scores) of zeaxanthin/lutein, β-cryptoxanthin, lycopene, α-carotene, β-carotene

c Comparison of 1, 2 or 3 times CES-D⩾16 with CES-D score 0 times ⩾16 (over CARDIA exam years 5, 10, 15)

d Number of self-reported somatic diseases included in count: high blood pressure, high cholesterol, heart problem, diabetes, hepatitis in the past year, kidney failure/dialysis/transplant in the past year, nephritis in the past year, other kidney disease in the past year, liver cirrhosis, other liver disease in the past year, gallstones in the past year, migraine in the past year, peripheral vascular disease, cancer (ever), thyroid disease (ever), ulcer in the past year, other digestive disease in the past year, gout in the past year, asthma in the past year, epilepsy with seizures in the past year, tuberculosis in the past year, emphysema in the past year, multiple sclerosis in the past year, stroke in the past year, chronic bronchitis in the past year, HIV (ever), blood clot (past year), other major disease, polycystic ovarian syndrome.

e Use of a multivitamin, vitamin A, C, E, beta-carotene or an antioxidant combination.

f Average score over CARDIA exam years 0, 7 and 20.

**Table 3:** Cross-sectional multivariable associations of F2-isoprostanes and carotenoids with depressive symptoms (CES-D)^a^

	*CARDIA exam year 15*		*Model 1*			*Model 2*			*Model 3*									
		*Age, sex, race, education*			*Age, sex, race,* *education,* *somatic disease,* *supplement use*			*Age, sex, race,* *education,* *somatic disease,* *supplement use,* *diet, BMI,* *smoking, alcohol,* *physical activity*										
		N	β	P	N	β	P	N	β	P								
*F2-isoprostanes*										
CES-D score		2968	0.05	0.009	2955	0.05	0.010	2926	0.01	0.731
CES-D⩾16		2968	0.06	0.002	2955	0.06	0.001	2926	0.03	0.113
CES-D⩾16 and/or AD use		2968	0.06	0.001	2955	0.06	0.001	2926	0.03	0.047
*N* CES-D ⩾16^b^	0	2592	Ref.		2582	Ref.		2558	Ref.	
	1		0.03	0.123		0.03	0.130		0.01	0.704
	2		0.05	0.009		0.05	0.008		0.03	0.111
	3		0.04	0.033		0.04	0.036		0.01	0.603
										
	*Sum of five carotenoids*^c^										
CES-D score		2865	−0.13	<0.001	2853	−0.13	<0.001	2825	−0.07	<0.001	
CES-D ⩾16		2865	−0.11	<0.001	2853	−0.11	<0.001	2825	−0.06	<0.001	
CES-D ⩾16 and/or AD use		2865	−0.11	<0.001	2853	−0.10	<0.001	2825	−0.06	<0.001	
*N* CES-D ⩾16^b^	0	2508	Ref.		2499			2475	Ref.		
	1		−0.03	0.083		−0.03	0.096		0.00	0.981	
	2		−0.07	0.001		−0.06	0.001		−0.02	0.206	
	3		−0.07	<0.001		−0.07	<0.001		−0.03	0.062	

Abbreviations: AD, antidepressant; BMI, body mass index; CARDIA, Coronary Artery Risk Development in Young Adults; CES-D, Center for Epidemiologic Studies Depression Scale; *N*, number; Ref., reference.

a F2-isoprostanes and carotenoids are log-transformed for linear regression analysis. Results are reported as standardized regression coefficients. All the results are adjusted for CENTER at baseline.

b Comparison of 1, 2 or 3 CES-D⩾16 with CES-D score 0⩾16 over years 5, 10, 15 as reference group.

c Sum of standardized values (*t*-scores) of zeaxanthin/lutein, β-cryptoxanthin, lycopene, α-carotene, β-carotene.

**Table 4:** Cross-lagged longitudinal multivariable analyses of F2-isoprostanes and carotenoids with depressive symptoms (CES-D)^a^

	*CARDIA exam years 10, 15 and 20*			*Model 1*			*Model 2*			*Model 3*										
*Independent variable*	*Dependent variable*	*Adjusted for*	*Age, sex, race,* *education*			*Age, sex, race*, *education*, *somatic disease*, *supplement use*			*Age, sex, race,* *education*, *somatic disease*, *supplement use*, *diet, BMI*, *smoking, alcohol*, *physical activity*											
			N	β	P	N	β	P	N	β	P									
*F2-isoprostanes*											
CES-D^b^	F2-isoprostanes 5 years later	Previous F2-isoprostane levels	2334	0.06	0.001	2321	0.05	0.002	2304	0.03	0.064
CES-D⩾16^b^	F2-isoprostanes 5 years later	Previous F2-isoprostane levels	2334	0.05	0.003	2321	0.05	0.004	2304	0.03	0.048
CES-D⩾16 and/or AD^b^	F2-isoprostanes 5 years later	Previous F2-isoprostane levels	2334	0.06	<0.001	2321	0.06	0.001	2304	0.04	0.012
F2-isoprostanes^c^	CES-D 5 years later	Previous CES-D scores	2589	0.04	0.028	2576	0.04	0.028	2506	0.03	0.095
										
	*Sum of five carotenoids*^d^											
CES-D^e^	Sum of five carotenoids 5 years later	Previous carotenoid levels	2356	−0.07	<0.001	2347	−0.07	<0.001	2306	−0.05	0.004	
CES-D⩾16^e^	Sum of five carotenoids 5 years later	Previous carotenoid levels	2356	−0.06	<0.001	2347	−0.06	<0.001	2306	−0.05	0.002	
CES-D⩾16 and/or AD^e^	Sum of five carotenoids 5 years later	Previous carotenoid levels	2356	−0.06	<0.001	2347	−0.06	<0.001	2306	−0.05	0.004	
Sum of five carotenoids^f^	CES-D 5 years later	Previous CES-D scores	2504	−0.03	0.069	2492	−0.03	0.086	2476	−0.01	0.560	

Abbreviations: AD, antidepressant; BMI, body mass index; CARDIA, Coronary Artery Risk Development in Young Adults; CES-D, Center for Epidemiologic Studies Depression Scale; *N*, number.

a F2-isoprostanes and carotenoids log-transformed for linear regression analysis. Results are reported as standardized regression coefficients. All the results are adjusted for CENTER at baseline.

b CES-D at year 15, F2-isoprostanes at year 20, covariates at year 15; adjusted for F2-isoprostanes at year 15.

c F2-isoprostanes at year 15, CES-D at year 20, covariates at year 15; adjusted for CES-D scores at year 15.

d Sum of standardized (*t*-scores) zeaxanthin/lutein, β-cryptoxanthin, lycopene, α-carotene, β-carotene.

e CES-D at year 10, sum of five carotenoids at year 15, covariates at year 10; adjusted for carotenoids at year 7 (as there are no data on carotenoids available in year 10 (see Figure 1)).

f Sum of five carotenoids at year 15, CES-D at year 20, covariates at year 15; adjusted for CES-D scores at year 15.

## References

[bib1] Vos T, Flaxman AD, Naghavi M, Lozano R, Michaud C, Ezzati M et al. Years lived with disability (YLDs) for 1160 sequelae of 289 diseases and injuries 1990-2010: a systematic analysis for the Global Burden of Disease Study 2010. Lancet 2012; 380: 2163–2196. 23245607 10.1016/S0140-6736(12)61729-2 PMC6350784

[bib2] Penninx BWJH, Milaneschi Y, Lamers F, Vogelzangs N. Understanding the somatic consequences of depression: biological mechanisms and the role of depression symptom profile. BMC Med 2013; 11: 129. 23672628 10.1186/1741-7015-11-129 PMC3661358

[bib3] Nicholson A, Kuper H, Hemingway H. Depression as an aetiologic and prognostic factor in coronary heart disease: a meta-analysis of 6362 events among 146 538 participants in 54 observational studies. Eur Heart J 2006; 27: 2763–2774. 17082208 10.1093/eurheartj/ehl338

[bib4] Luppino FS, de Wit LM, Bouvy PF, Stijnen T, Cuijpers P, Penninx BWJH, Zitman FG. Overweight, obesity, and depression: a systematic review and meta-analysis of longitudinal studies. Arch Gen Psychiatry 2010; 67: 220–229. 20194822 10.1001/archgenpsychiatry.2010.2

[bib5] Mezuk B, Eaton WW, Albrecht S, Golden SH. Depression and type 2 diabetes over the lifespan: a meta-analysis. Diabetes Care 2008; 31: 2383–2390. 19033418 10.2337/dc08-0985 PMC2584200

[bib6] Chida Y, Hamer M, Wardle J, Steptoe A. Do stress-related psychosocial factors contribute to cancer incidence and survival? Nat Clin Pract Oncol 2008; 5: 466–475. 18493231 10.1038/ncponc1134

[bib7] Barnes DE, Alexopoulos GS, Lopez OL, Williamson JD, Yaffe K. Depressive symptoms, vascular disease, and mild cognitive impairment: findings from the Cardiovascular Health Study. Arch Gen Psychiatry 2006; 63: 273–279. 16520432 10.1001/archpsyc.63.3.273

[bib8] Cuijpers P, Smit F. Excess mortality in depression: a meta-analysis of community studies. J Affect Disord 2002; 72: 227–236. 12450639 10.1016/s0165-0327(01)00413-x

[bib9] Valko M, Leibfritz D, Moncol J, Cronin MTD, Mazur M, Telser J. Free radicals and antioxidants in normal physiological functions and human disease. Int J Biochem Cell Biol 2007; 39: 44–84. 16978905 10.1016/j.biocel.2006.07.001

[bib10] Black CN, Bot M, Scheffer PG, Cuijpers P, Penninx BWJH. Is depression associated with increased oxidative stress? A systematic review and meta-analysis. Psychoneuroendocrinology 2014; 51C: 164–175. 10.1016/j.psyneuen.2014.09.025 25462890

[bib11] Dalle-Donne I, Rossi R, Colombo R, Giustarini D, Milzani A. Biomarkers of oxidative damage in human disease. Clin Chem 2006; 52: 601–623. 16484333 10.1373/clinchem.2005.061408

[bib12] Janicki-Deverts D, Cohen S, Matthews KA, Gross MD, Jacobs DRJ. Socioeconomic status, antioxidant micronutrients, and correlates of oxidative damage: the Coronary Artery Risk Development in Young Adults (CARDIA) study. Psychosom Med 2009; 71: 541–548. 19414620 10.1097/PSY.0b013e31819e7526 PMC3925505

[bib13] Glassman AH, Helzer JE, Covey LS, Cottler LB, Stetner F, Tipp JE et al. Smoking, smoking cessation, and major depression. JAMA 1990; 264: 1546–1549. 2395194

[bib14] Abu-Omar K, Rutten A, Lehtinen V. Mental health and physical activity in the European Union. Soz Praventivmed 2004; 49: 301–309. 15497649 10.1007/s00038-004-3109-8

[bib15] Sullivan LE, Fiellin DA, O'Connor PG. The prevalence and impact of alcohol problems in major depression: a systematic review. Am J Med 2005; 118: 330–341. 15808128 10.1016/j.amjmed.2005.01.007

[bib16] Palta P, Samuel LJ, Miller ER 3rd, Szanton SL. Depression and oxidative stress: results from a meta-analysis of observational studies. Psychosom Med 2014; 76: 12–19. 24336428 10.1097/PSY.0000000000000009 PMC4290164

[bib17] Beydoun MA, Beydoun HA, Boueiz A, Shroff MR, Zonderman AB. Antioxidant status and its association with elevated depressive symptoms among US adults: National Health and Nutrition Examination Surveys 2005-6. Br J Nutr 2013; 109: 1714–1729. 22935166 10.1017/S0007114512003467 PMC3810278

[bib18] Milaneschi Y, Bandinelli S, Penninx BW, Corsi AM, Lauretani F, Vazzana R et al. The relationship between plasma carotenoids and depressive symptoms in older persons. World J Biol Psychiatry 2012; 13: 588–598. 21929378 10.3109/15622975.2011.597876 PMC3360996

[bib19] Sarandol A, Sarandol E, Eker SS, Erdinc S, Vatansever E, Kirli S. Major depressive disorder is accompanied with oxidative stress: short-term antidepressant treatment does not alter oxidative-antioxidative systems. Hum Psychopharmacol 2007; 22: 67–73. 17299810 10.1002/hup.829

[bib20] Niki E. Biomarkers of lipid peroxidation in clinical material. Biochim Biophys Acta 2014; 1840: 809–817. 23541987 10.1016/j.bbagen.2013.03.020

[bib21] Milne GL, Dai Q, Roberts LJ. The isoprostanes-25 years later. Biochim Biophys Acta 2015; 1851: 433–445. 25449649 10.1016/j.bbalip.2014.10.007 PMC5404383

[bib22] Kadiiska MB, Gladen BC, Baird DD, Germolec D, Graham LB, Parker CE et al. Biomarkers of oxidative stress study II: are oxidation products of lipids, proteins, and DNA markers of CCl4 poisoning? Free Radic Biol Med 2005; 38: 698–710. 15721980 10.1016/j.freeradbiomed.2004.09.017

[bib23] Milne GL, Yin H, Brooks JD, Sanchez S, Jackson Roberts L, Morrow JD. Quantification of F2-isoprostanes in biological fluids and tissues as a measure of oxidant stress. Methods Enzymol 2007; 433: 113–126. 17954231 10.1016/S0076-6879(07)33006-1

[bib24] Ford ES, Mokdad AH, Giles WH, Brown DW. The metabolic syndrome and antioxidant concentrations: findings from the Third National Health and Nutrition Examination Survey. Diabetes 2003; 52: 2346–2352. 12941775 10.2337/diabetes.52.9.2346

[bib25] Beydoun MA, Shroff MR, Chen X, Beydoun HA, Wang Y, Zonderman AB. Serum antioxidant status is associated with metabolic syndrome among U.S. adults in recent national surveys. J Nutr 2011; 141: 903–913. 21451127 10.3945/jn.110.136580 PMC3077890

[bib26] Coyne T, Ibiebele TI, Baade PD, Dobson A, McClintock C, Dunn S et al. Diabetes mellitus and serum carotenoids: findings of a population-based study in Queensland, Australia. Am J Clin Nutr 2005; 82: 685–693. 16155284 10.1093/ajcn.82.3.685

[bib27] Ford ES, Will JC, Bowman BA, Narayan KM. Diabetes mellitus and serum carotenoids: findings from the Third National Health and Nutrition Examination Survey. Am J Epidemiol 1999; 149: 168–176. 9921962 10.1093/oxfordjournals.aje.a009783

[bib28] Hozawa A, Jacobs DR, Steffes MW, Gross MD, Steffen LM, Lee D-H. Associations of serum carotenoid concentrations with the development of diabetes and with insulin concentration: interaction with smoking: the Coronary Artery Risk Development in Young Adults (CARDIA) Study. Am J Epidemiol 2006; 163: 929–937. 16597706 10.1093/aje/kwj136

[bib29] Sesso HD, Buring JE, Norkus EP, Gaziano JM. Plasma lycopene, other carotenoids, and retinol and the risk of cardiovascular disease in women. Am J Clin Nutr 2004; 79: 47–53. 14684396 10.1093/ajcn/79.1.47

[bib30] Osganian SK, Stampfer MJ, Rimm E, Spiegelman D, Manson JE, Willett WC. Dietary carotenoids and risk of coronary artery disease in women. Am J Clin Nutr 2003; 77: 1390–1399. 12791615 10.1093/ajcn/77.6.1390

[bib31] Eliassen AH, Liao X, Rosner B, Tamimi RM, Tworoger SS, Hankinson SE. Plasma carotenoids and risk of breast cancer over 20 y of follow-up. Am J Clin Nutr 2015; 101: 1197–1205. 25877493 10.3945/ajcn.114.105080 PMC4441811

[bib32] Jomova K, Valko M. Health protective effects of carotenoids and their interactions with other biological antioxidants. Eur J Med Chem 2013; 70: 102–110. 24141200 10.1016/j.ejmech.2013.09.054

[bib33] Fiedor J, Burda K. Potential role of carotenoids as antioxidants in human health and disease. Nutrients 2014; 6: 466–488. 24473231 10.3390/nu6020466 PMC3942711

[bib34] Meyer KA, Sijtsma FPC, Nettleton JA, Steffen LM, Horn L, Van, Shikany JM et al. Dietary patterns are associated with plasma F2-isoprostanes in an observational cohort study of adults. Free Radic Biol Med 2014; 57: 1–20. 10.1016/j.freeradbiomed.2012.08.574 PMC3872789 22982044

[bib35] Moylan S, Maes M, Wray NR, Berk M. The neuroprogressive nature of major depressive disorder: pathways to disease evolution and resistance, and therapeutic implications. Mol Psychiatry 2013; 18: 595–606. 22525486 10.1038/mp.2012.33

[bib36] Friedman GD, Cutter GR, Donahue RP, Hughes GH, Hulley SB, Jacobs DR et al. CARDIA: study design, recruitment, and some characteristics of the examined subjects. J Clin Epidemiol 1988; 41: 1105–1116. 3204420 10.1016/0895-4356(88)90080-7

[bib37] Radloff LS. The CES-D scale: a self-report depression scale for research in the general population. Appl Psychol Meas 1977; 1: 385–401.

[bib38] Shafer AB. Meta-analysis of the factor structures of four depression questionnaires: Beck, CES-D, Hamilton, and Zung. J Clin Psychol 2006; 62: 123–146. 16287149 10.1002/jclp.20213

[bib39] Morrow JD, Roberts LJ 2nd. Mass spectrometric quantification of F2-isoprostanes in biological fluids and tissues as measure of oxidant stress. Methods Enzymol 1999; 300: 3–12. 9919502 10.1016/s0076-6879(99)00106-8

[bib40] Gross M, Steffes M, Jacobs DR, Yu X, Lewis L, Lewis CE et al. Plasma F2-isoprostanes and coronary artery calcification: the CARDIA study. Clin Chem 2005; 51: 125–131. 15514100 10.1373/clinchem.2004.037630

[bib41] Bieri JG, Brown ED, Smith JC. Determination of individual carotenoids in human-plasma by high-performance liquid-chromatography. J Liq Chromatogr 1985; 8: 473–484.

[bib42] Craft NE, Brown ED, Smith JCJ. Effects of storage and handling conditions on concentrations of individual carotenoids, retinol, and tocopherol in plasma. Clin Chem 1988; 34: 44–48. 3338183

[bib43] Gross MD, Prouty CB, Jacobs DRJ. Stability of carotenoids and alpha-tocopherol during blood collection and processing procedures. Clin Chem 1995; 41: S943–S944. 7768018

[bib44] Iribarren C, Folsom AR, Jacobs DRJ, Gross MD, Belcher JD, Eckfeldt JH. Association of serum vitamin levels, LDL susceptibility to oxidation, and autoantibodies against MDA-LDL with carotid atherosclerosis. A case-control study. The ARIC Study Investigators. Atherosclerosis risk in communities. Arterioscler Thromb Vasc Biol 1997; 17: 1171–1177. 9194770 10.1161/01.atv.17.6.1171

[bib45] Hozawa A, Jacobs DR, Steffes MW, Gross MD, Steffen LM, Lee D-H. Relationships of circulating carotenoid concentrations with several markers of inflammation, oxidative stress, and endothelial dysfunction: the Coronary Artery Risk Development in Young Adults (CARDIA)/Young Adult Longitudinal Trends in Antioxidants (YALT. Clin Chem 2007; 53: 447–455. 17234732 10.1373/clinchem.2006.074930 PMC2440581

[bib46] Beydoun MA, Fanelli-Kuczmarski MT, Kitner-Triolo MH, Beydoun HA, Kaufman JS, Mason MA et al. Dietary antioxidant intake and its association with cognitive function in an ethnically diverse sample of US adults. Psychosom Med 2015; 77: 68–82. 25478706 10.1097/PSY.0000000000000129 PMC4597309

[bib47] Jacobs DRJ P, Hahn LPM, Haskell WLP, Pirie PP, Sidney SM. Validity and reliability of short physical activity history: CARDIA and the Minnesota Heart Health Program. J Cardiopulm Rehabil 1989; 9: 448–459. 10.1097/00008483-198911000-00003 PMC5894828 29657358

[bib48] Cohen J. Statistical Power Analysis for the Behavioral Sciences. 2nd edn. Lawrence Erlbaum Associates: Hillsdale, NJ, USA, 1988.

[bib49] McMartin SE, Jacka FN, Colman I. The association between fruit and vegetable consumption and mental health disorders: evidence from five waves of a national survey of Canadians. Prev Med 2013; 56: 225–230. 23295173 10.1016/j.ypmed.2012.12.016

[bib50] Payne ME, Steck SE, George RR, Steffens DC. Fruit, vegetable, and antioxidant intakes are lower in older adults with depression. J Acad Nutr Diet 2012; 112: 2022–2027. 23174689 10.1016/j.jand.2012.08.026 PMC3520090

[bib51] Aseervatham GSB, Sivasudha T, Jeyadevi R, Arul Ananth D. Environmental factors and unhealthy lifestyle influence oxidative stress in humans—an overview. Environ Sci Pollut Res Int 2013; 20: 4356–4369. 23636598 10.1007/s11356-013-1748-0

[bib52] Alberg A. The influence of cigarette smoking on circulating concentrations of antioxidant micronutrients. Toxicology 2002; 180: 121–137. 12324189 10.1016/s0300-483x(02)00386-4

[bib53] Handelman GJ, Packer L, Cross CE. Destruction of tocopherols, carotenoids, and retinol in human plasma by cigarette smoke. Am J Clin Nutr 1996; 63: 559–565. 8599320 10.1093/ajcn/63.4.559

[bib54] Beydoun MA, Nalls MA, Canas JA, Evans MK, Zonderman AB. Gene polymorphisms and gene scores linked to low serum carotenoid status and their associations with metabolic disturbance and depressive symptoms in African-American adults. Br J Nutr 2014; 112: 992–1003. 25201307 10.1017/S0007114514001706 PMC4887136

[bib55] Hroudová J, Fišar Z. Connectivity between mitochondrial functions and psychiatric disorders. Psychiatry Clin Neurosci 2011; 65: 130–141. 21414088 10.1111/j.1440-1819.2010.02178.x

[bib56] Lee S-Y, Lee S-J, Han C, Patkar AA, Masand PS, Pae C-U. Oxidative/nitrosative stress and antidepressants: targets for novel antidepressants. Prog Neuropsychopharmacol Biol Psychiatry 2013; 46: 224–235. 23022673 10.1016/j.pnpbp.2012.09.008

[bib57] Khanzode SD, Dakhale GN, Khanzode SS, Saoji A, Palasodkar R. Oxidative damage and major depression: the potential antioxidant action of selective serotonin re-uptake inhibitors. Redox Rep 2003; 8: 365–370. 14980069 10.1179/135100003225003393

[bib58] Galecki P, Szemraj J, Bienkiewicz M, Zboralski K, Galecka E. Oxidative stress parameters after combined fluoxetine and acetylsalicylic acid therapy in depressive patients. Hum Psychopharmacol 2009; 24: 277–286. 19319921 10.1002/hup.1014

[bib59] Herken H, Gurel A, Selek S, Armutcu F, Ozen ME, Bulut M et al. Adenosine deaminase, nitric oxide, superoxide dismutase, and xanthine oxidase in patients with major depression: impact of antidepressant treatment. Arch Med Res 2007; 38: 247–252. 17227736 10.1016/j.arcmed.2006.10.005

[bib60] Cumurcu BE, Ozyurt H, Etikan I, Demir S, Karlidag R. Total antioxidant capacity and total oxidant status in patients with major depression: impact of antidepressant treatment. Psychiatry Clin Neurosci 2009; 63: 639–645. 19674383 10.1111/j.1440-1819.2009.02004.x

[bib61] Kotan VO, Sarandol E, Kirhan E, Ozkaya G, Kirli S. Effects of long-term antidepressant treatment on oxidative status in major depressive disorder: a 24-week follow-up study. Prog Neuropsychopharmacol Biol Psychiatry 2011; 35: 1284–1290. 21515329 10.1016/j.pnpbp.2011.03.021

[bib62] Bilici M, Efe H, Koroglu MA, Uydu HA, Bekaroglu M, Deger O. Antioxidative enzyme activities and lipid peroxidation in major depression: alterations by antidepressant treatments. J Affect Disord 2001; 64: 43–51. 11292519 10.1016/s0165-0327(00)00199-3

[bib63] Maes M, Mihaylova I, Kubera M, Uytterhoeven M, Vrydags N, Bosmans E. Lower plasma Coenzyme Q10 in depression: a marker for treatment resistance and chronic fatigue in depression and a risk factor to cardiovascular disorder in that illness. Neuro Endocrinol Lett 2009; 30: 462–469. 20010493

[bib64] Chung CP, Schmidt D, Stein CM, Morrow JD, Salomon RM. Increased oxidative stress in patients with depression and its relationship to treatment. Psychiatry Res 2012; 206: 213–216. 23245537 10.1016/j.psychres.2012.10.018 PMC3615036

[bib65] Rawdin BS, Mellon SH, Dhabhar FS, Epel ES, Puterman E, Su Y et al. Dysregulated relationship of inflammation and oxidative stress in major depression. Brain Behav Immun 2012; 31: 143–152. 23201587 10.1016/j.bbi.2012.11.011 PMC3669232

[bib66] Gawryluk JW, Wang J-F, Andreazza AC, Shao L, Yatham LN, Young LT. Prefrontal cortex glutathione S-transferase levels in patients with bipolar disorder, major depression and schizophrenia. Int J Neuropsychopharmacol 2011; 14: 1069–1074. 21733244 10.1017/S1461145711000617

[bib67] Gawryluk JW, Wang J-F, Andreazza AC, Shao L, Young LT. Decreased levels of glutathione, the major brain antioxidant, in post-mortem prefrontal cortex from patients with psychiatric disorders. Int J Neuropsychopharmacol 2011; 14: 123–130. 20633320 10.1017/S1461145710000805

[bib68] Bjelakovic G, Nikolova D, Gluud LL, Simonetti RG, Gluud C. Antioxidant supplements for prevention of mortality in healthy participants and patients with various diseases. Cochrane Database Syst Rev 2012; 3: CD007176. 22419320 10.1002/14651858.CD007176.pub2 PMC8407395

[bib69] Bjelakovic G, Nikolova D, Simonetti RG, Gluud C. Antioxidant supplements for preventing gastrointestinal cancers. Cochrane Database Syst Rev 2008; CD004183. 18677777 10.1002/14651858.CD004183.pub3 PMC12276870

[bib70] Cortés-Jofré M, Rueda J-R, Corsini-Muñoz G, Fonseca-Cortés C, Caraballoso M, Bonfill Cosp X. Drugs for preventing lung cancer in healthy people. Cochrane Database Syst Rev 2012; 10: CD002141. 23076895 10.1002/14651858.CD002141.pub2

[bib71] Satia JA, Littman A, Slatore CG, Galanko JA, White E. Long-term use of beta-carotene, retinol, lycopene, and lutein supplements and lung cancer risk: results from the VITamins And Lifestyle (VITAL) study. Am J Epidemiol 2009; 169: 815–828. 19208726 10.1093/aje/kwn409 PMC2842198

[bib72] Tanvetyanon T, Bepler G. Beta-carotene in multivitamins and the possible risk of lung cancer among smokers versus former smokers: a meta-analysis and evaluation of national brands. Cancer 2008; 113: 150–157. 18429004 10.1002/cncr.23527

[bib73] Grosso G, Pajak A, Marventano S, Castellano S, Galvano F, Bucolo C et al. Role of omega-3 fatty acids in the treatment of depressive disorders: a comprehensive meta-analysis of randomized clinical trials. PLoS One 2014; 9: e96905. 24805797 10.1371/journal.pone.0096905 PMC4013121

[bib74] Lai JS, Hiles S, Bisquera A, Hure AJ, McEvoy M, Attia J. A systematic review and meta-analysis of dietary patterns and depression in community-dwelling adults. Am J Clin Nutr 2014; 99: 181–197. 24196402 10.3945/ajcn.113.069880

