# Supplementary material for: Oxidative stress, anti-oxidants and the cross-sectional and longitudinal association with depressive symptoms: results from the CARDIA study
Source: Transl Psychiatry. 2016 Feb 23;6(2):e743–. doi: 10.1038/tp.2016.5 (PMC4872434; doi:10.1038/tp.2016.5)
Supplement: Supplementary Table 1 [file tp20165x1.doc]

| **Supplemental Table 1. Cross-sectional univariate associations of carotenoids with covariates ^a^**  *CARDIA exam year 15* | | | | | | | | | | | |
| --- | --- | --- | --- | --- | --- | --- | --- | --- | --- | --- | --- |
|  | | **Zeaxanthin/lutein** | | **β-cryptoxanthin** | | **Lycopene** | | **α-carotene** | | **β-carotene** | |
|  |  | **β** | **p** | **β** | **p** | **β** | **p** | **β** | **p** | **β** | **p** |
| **Depressive symptoms** |  |  |  |  |  |  |  |  |  |  |  |
| CES-D |  | -.13 | <.001 | -.12 | <.001 | -.09 | <.001 | -.17 | <.001 | -.16 | <.001 |
| CES-D ≥ 16 |  | -.11 | <.001 | -.09 | <.001 | -.08 | <.001 | -.14 | <.001 | -.14 | <.001 |
| CES-D ≥ 16 and/or antidepressant use | | -.12 | <.001 | -.10 | <.001 | -.07 | <.001 | -.11 | <.001 | -.11 | <.001 |
| Antidepressant users |  | -.11 | <.001 | -.08 | <.001 | -.07 | <.001 | -.01 | .465 | -.01 | .679 |
|  |  |  |  |  |  |  |  |  |  |  |  |
| *N* of times CES-D ≥ 16 | 0 |  | Ref. |  | Ref. |  | Ref. |  | Ref. |  | Ref. |
| over years 0, 10 and 15 ^b^ | 1 | -.06 | .003 | -.05 | .012 | -.02 | .229 | -.08 | <.001 | -.06 | .001 |
|  | 2 | -.05 | .020 | -.03 | .082 | -.06 | .005 | -.10 | <.001 | -.10 | <.001 |
|  | 3 | -.09 | <.001 | -.07 | <.001 | -.06 | .002 | -.10 | <.001 | -.10 | <.001 |
| **Socio-demographics** |  |  |  |  |  |  |  |  |  |  |  |
| Age |  | .05 | .012 | .00 | .909 | -.06 | .003 | .09 | <.001 | .09 | <.001 |
| Sex | (male reference) | -.05 | .012 | .01 | .680 | -.09 | <.001 | .09 | <.001 | .13 | <.001 |
| Race | (white reference) | -.03 | .177 | .02 | .375 | .00 | .932 | -.27 | <.001 | -.20 | <.001 |
|  |  |  |  |  |  |  |  |  |  |  |  |
| Education | ≤high school |  | Ref. |  | Ref. |  | Ref. |  | Ref. |  | Ref. |
|  | (some) college | .18 | <.001 | .17 | <.001 | .02 | .252 | .25 | <.001 | .22 | <.001 |
|  | ≥ master’s degree | .07 | <.001 | .07 | <.001 | .03 | .128 | .08 | <.001 | .07 | <.001 |
| **Health and lifestyle** |  |  |  |  |  |  |  |  |  |  |  |
| *N* somatic diseases ^c^ |  | -.06 | .002 | -.10 | <.001 | -.06 | .001 | -.04 | .052 | -,06 | .002 |
|  |  |  |  |  |  |  |  |  |  |  |  |
| Supplement users ^d^ |  | .04 | .033 | .08 | <.001 | -.01 | .511 | .16 | <.001 | .21 | <.001 |
|  |  |  |  |  |  |  |  |  |  |  |  |
| Diet quality score ^e^ |  | .27 | <.001 | .20 | <.001 | .00 | .858 | .43 | <.001 | .38 | <.001 |
|  |  |  |  |  |  |  |  |  |  |  |  |
| Smoker | Non |  | Ref. |  | Ref. |  | Ref. |  | Ref. |  | Ref. |
|  | Current | -.17 | <.001 | -.23 | <.001 | -.05 | .011 | -.26 | <.001 | -.24 | <.001 |
|  |  |  |  |  |  |  |  |  |  |  |  |
| Alcohol | ♂/♀ 0 units/week |  | Ref. |  | Ref. |  | Ref. |  | Ref. |  | Ref. |
|  | ♂≤14 /♀≤7 units/week | .09 | <.001 | .08 | <.001 | .07 | <.001 | .06 | <.001 | .04 | .029 |
|  | ♂>14/♀>7 units/week | .03 | .108 | -.09 | <.001 | .00 | .882 | -.04 | .055 | -.08 | <.001 |
|  |  |  |  |  |  |  |  |  |  |  |  |
| BMI kg/m­^2^ |  | -.21 | <.001 | -.20 | <.001 | -.02 | .400 | -.28 | <.001 | -.31 | <.001 |
|  |  |  |  |  |  |  |  |  |  |  |  |
| Physical activity | exercise units (EU) | .10 | <.001 | .09 | <.001 | .04 | .046 | .11 | <.001 | .12 | <.001 |

AD= antidepressant; CES-D= Center for Epidemiologic Studies Depression Scale; *N*= number; BMI= body mass index.

^a^ Carotenoids log transformed for linear regression analysis. Results are reported as standardized regression coefficients. All results are adjusted for CENTER at baseline.

^b^ Comparison of 1, 2 or 3 times CES-D ≥16 with CES-D score 0 times ≥16 (over years 5, 10, 15).

^c^ Number of self-reported somatic diseases included in count: high blood pressure, high cholesterol, heart problem, diabetes, hepatitis in past year, kidney failure/dialysis/transplant past year, nephritis in past year, other kidney disease past year, liver cirrhosis, other liver disease past year, gallstones in past year, migraine in past year, peripheral vascular disease, cancer (ever), thyroid disease (ever), ulcer past year, other digestive disease past year, gout past year, asthma past year, epilepsy with seizures past year, tuberculosis past year, emphysema past year, multiple sclerosis past year, stroke past year, chronic bronchitis past year, HIV (ever), blood clot (past year), other major disease, poly cystic ovarian syndrome.

^d^ Use of a multivitamin, vitamin A, C, E, beta-carotene or an anti-oxidant combination.

^e^ Average score over CARDIA exam years 0, 7 and 20.
