# Supplementary material for: Oxidative stress, anti-oxidants and the cross-sectional and longitudinal association with depressive symptoms: results from the CARDIA study
Source: Transl Psychiatry. 2016 Feb 23;6(2):e743–. doi: 10.1038/tp.2016.5 (PMC4872434; doi:10.1038/tp.2016.5)
Supplement: Supplementary Table 2 [file tp20165x2.doc]

| **Supplemental Table 2. Cross-sectional multivariable associations of carotenoids with**  **depressive symptoms (CES-D) ^a^**  *CARDIA exam year 15* | | | | | | | | | | |
| --- | --- | --- | --- | --- | --- | --- | --- | --- | --- | --- |
|  | | **Model 1** | | | **Model 2** | | | **Model 3** | | |
|  | | Age, Sex, Race | | | Age, Sex, Race | | | Age, Sex, Race | | |
|  | | Education | | | Education | | | Education | | |
|  | |  | | | Somatic disease | | | Somatic disease | | |
|  | |  |  |  | Supplement use | | | Supplement use | | |
|  | |  |  |  |  | | | Diet, BMI | | |
|  | |  |  |  |  |  |  | Smoking, Alcohol | | |
|  | |  |  |  |  |  |  | Physical activity | | |
|  |  |  |  |  |  |  |  |  |  |  |
|  | | ***N*** | **β** | **p** | ***N*** | **β** | **p** | ***N*** | **β** | **p** |
| **Zeaxanthin/lutein** | |  |  | |  |  | |  |  | |
| CES-D score | | 2883 | -.11 | <.001 | 2871 | -.11 | <.001 | 2843 | -.07 | <.001 |
| CES-D ≥ 16 | | 2883 | -.10 | <.001 | 2871 | -.10 | <.001 | 2843 | -.07 | <.001 |
| CES-D ≥ 16 and/or AD use | | 2883 | -.11 | <.001 | 2871 | -.11 | <.001 | 2843 | -.08 | <.001 |
| *N* CES-D ≥ 16 ^b^ | 1 | 2524 | -.05 | .021 | 2515 | -.04 | .029 | 2491 | -.01 | .439 |
| 2 | |  | -.03 | .083 |  | -.03 | .113 |  | -.01 | .767 |
| 3 | |  | -.07 | <.001 |  | -.07 | <.001 |  | -.05 | .015 |
| **β-cryptoxanthin** | |  |  |  |  |  |  |  |  |  |
| CES-D score | | 2881 | -.11 | <.001 | 2869 | -.10 | <.001 | 2841 | -.04 | .015 |
| CES-D ≥ 16 | | 2881 | -.09 | <.001 | 2869 | -.08 | <.001 | 2841 | -.03 | .096 |
| CES-D ≥ 16 and/or AD use | | 2881 | -.09 | <.001 | 2869 | -.08 | <.001 | 2841 | -.04 | .028 |
| *N* CES-D ≥ 16 ^b^ | 1 | 2522 | -.04 | .035 | 2513 | -.04 | .054 | 2489 | -.01 | .680 |
| 2 | |  | -.04 | .067 |  | -.03 | .104 |  | .01 | .522 |
| 3 | |  | -.07 | <.001 |  | -.07 | .001 |  | -.03 | .158 |
| **Lycopene** | |  |  |  |  |  |  |  |  |  |
| CES-D score | | 2883 | -.08 | <.001 | 2871 | -.08 | <.001 | 2843 | -.07 | .001 |
| CES-D ≥ 16 | | 2883 | -.07 | <.001 | 2871 | -.07 | <.001 | 2843 | -.06 | .002 |
| CES-D ≥ 16 and/or AD use | | 2883 | -.06 | .002 | 2871 | -.05 | .007 | 2843 | -.05 | .021 |
| *N* CES-D ≥ 16 ^b^ | 1 | 2524 | -.02 | .450 | 2515 | -.01 | .488 | 2491 | -.01 | .727 |
| 2 | |  | -.05 | .009 |  | -.05 | .014 |  | -.05 | .030 |
| 3 | |  | -.06 | .004 |  | -.06 | .006 |  | -.05 | .014 |
| **α-carotene** | |  |  |  |  |  |  |  |  |  |
| CES-D score | | 2866 | -.13 | <.001 | 2854 | -.13 | <.001 | 2826 | -.06 | <.001 |
| CES-D ≥ 16 | | 2866 | -.10 | <.001 | 2854 | -.10 | <.001 | 2826 | -.05 | .001 |
| CES-D ≥ 16 and/or AD use | | 2866 | -.09 | <.001 | 2854 | -.09 | <.001 | 2826 | -.05 | .002 |
| *N* CES-D ≥ 16 ^b^ | 1 | 2509 | -.06 | .001 | 2500 | -.06 | .001 | 2476 | -.03 | .135 |
| 2 | |  | -.05 | .004 |  | -.05 | .004 |  | -.01 | .414 |
| 3 | |  | -.06 | <.001 |  | -.06 | <.001 |  | -.03 | .140 |
| **β-carotene** | |  |  |  |  |  |  |  |  |  |
| CES-D score | | 2883 | -.14 | <.001 | 2871 | -.13 | <.001 | 2843 | -.07 | <.001 |
| CES-D ≥ 16 | | 2883 | -.11 | <.001 | 2871 | -.11 | <.001 | 2843 | -.06 | <.001 |
| CES-D ≥ 16 and/or AD use | | 2883 | -.10 | <.001 | 2871 | -.09 | <.001 | 2843 | -.05 | .003 |
| *N* CES-D ≥ 16 ^b^ | 1 | 2524 | -.05 | .007 | 2515 | -.05 | .007 | 2491 | -.02 | .309 |
| 2 | |  | -.07 | <.001 |  | -.07 | <.001 |  | -.03 | .108 |
| 3 | |  | -.08 | <.001 |  | -.08 | <.001 |  | -.04 | .025 |

AD= antidepressant; CES-D= Center for Epidemiologic Studies Depression Scale; *N*= number.

^a^ Carotenoids are log transformed for linear regression analysis. Results are reported as standardized regression coefficients. All results are adjusted for CENTER at baseline.

^b^ comparison of 1, 2 or 3 CES-D ≥16 with CES-D score 0 ≥16 over years 5, 10, 15.
