# Supplementary material for: Oxidative stress, anti-oxidants and the cross-sectional and longitudinal association with depressive symptoms: results from the CARDIA study
Source: Transl Psychiatry. 2016 Feb 23;6(2):e743–. doi: 10.1038/tp.2016.5 (PMC4872434; doi:10.1038/tp.2016.5)
Supplement: Supplementary Table 3 [file tp20165x3.doc]

| **Supplemental Table 3. Analyses of the % of change in estimate of depressive symptoms (CES-D) for F2-isoprostanes / carotenoids following the addition of health- and lifestyle factors ^a^**  *CARDIA exam year 15* | | | |
| --- | --- | --- | --- |
| **F2-isoprotanes (dependent variable )** | **β^c^** | **p** | **% change** |
|  |  |  |  |
| CES-D + sociodemographics | .047 | .009 |  |
|  |  |  |  |
| + BMI | .030 | .073 | - 36.2% |
| + smoke | .034 | .061 | - 27.7% |
| + diet | .036 | .038 | - 23.4% |
| + alcohol | .038 | .030 | - 19.1% |
| + physical activity | .039 | .029 | -17.0% |
| + somatic disease | .043 | .016 | - 10.6% |
| + supplement use | .049 | .006 | + 4.3% |
|  |  |  |  |
| **Sum of 5 carotenoids^b^  (dependent variable )** | **β** | **p** | **% change** |
|  |  |  |  |
| CES-D + sociodemographics | -.130 | <.001 |  |
|  |  |  |  |
| + smoke | -.099 | <.001 | - 23.8% |
| + diet | -.113 | <.001 | - 13.1% |
| + BMI | -.120 | <.001 | - 7.7% |
| + physical activity | -.121 | <.001 | - 6.9% |
| + somatic disease | -.123 | <.001 | - 5.4% |
| + alcohol | -.125 | <.001 | - 3.8% |
| + supplement use | -.132 | <.001 | + 1.5% |
|  |  |  |  |

^a^ F2-isoprostanes and carotenoids are log transformed for linear regression analysis. Results are reported as standardized regression coefficients. All results are adjusted for CENTER at baseline.

^b^ Sum of standardized values (t-scores) of zeaxanthin/lutein, β-cryptoxanthin, lycopene, α-carotene, β-carotene.

^c^ standardized regression coefficient of CES-D score
